# Health Literacy, Digital Health Literacy, and COVID-19 Pandemic Attitudes and Behaviors in U.S. College Students: Implications for Interventions

**DOI:** 10.3390/ijerph18063301

**Published:** 2021-03-23

**Authors:** Uday Patil, Uliana Kostareva, Molly Hadley, Jennifer A. Manganello, Orkan Okan, Kevin Dadaczynski, Philip M. Massey, Joy Agner, Tetine Sentell

**Affiliations:** 1 Office of Public Health Studies, Thompson School of Social Work and Public Health, University of Hawai‘i at Mānoa, 1960 East West Road, Honolulu, HI 96822, USA; tsentell@hawaii.edu; 2 School of Nursing and Dental Hygiene, University of Hawai‘i at Mānoa, 2528 McCarthy Mall, Honolulu, HI 96822, USA; uliana@hawaii.edu; 3 School of Public Health, University at Albany, One University Place, Rensselaer, NY 12144, USA; mhadley@albany.edu (M.H.); jmanganello@albany.edu (J.A.M.); 4 Interdisciplinary Centre for Health Literacy, Bielefeld University, Universitätsstraße 25, 33615 Bielefeld, Germany; orkan.okan@uni-bielefeld.de; 5 Department of Nursing and Health Science, Fulda University of Applied Sciences, Leipziger Straße 123, 36037 Fulda, Germany; kevin.dadaczynski@pg.hs-fulda.de; 6 Center for Applied Health Science, Leuphana University of Lueneburg, Wilschenbrucher Weg 84a, 21335 Lüneburg, Germany; 7 Department of Community Health and Prevention, Dornsife School of Public Health, Drexel University, 3215 Market St., Philadelphia, PA 19104, USA; pmm85@drexel.edu; 8 Department of Psychology, University of Hawai‘i at Mānoa, 2530 Dole Street, Sakamaki C400, Honolulu, HI 96822, USA; joyagner@hawaii.edu

**Keywords:** COVID-19, coronavirus, health literacy, digital health literacy, eHealth literacy, college student, online survey, social network

## Abstract

The COVID-19 pandemic has been accompanied by rapidly emerging evidence, changing guidance, and misinformation, which present new challenges for health literacy (HL) and digital health literacy (DHL) skills. This study explored whether COVID-19-related information access, attitudes, and behaviors were associated with health literacy and digital health literacy among college students in the United States. Self-reported measures of health literacy, along with items on pandemic-related attitudes, behaviors, information sources, and social networks, were collected online using a managed research panel. In July 2020, 256 responses were collected, which mirrored the racial/ethnic and gender diversity of U.S. colleges. Only 49% reported adequate HL, and 57% found DHL tasks easy overall. DHL did not vary by HL level. In multivariable models, both HL and DHL were independently associated with overall compliance with basic preventive practices. Higher DHL, but not HL, was significantly associated with greater willingness to get a COVID-19 vaccine and the belief that acquiring the disease would negatively impact their life. On average, respondents discussed health with 4–5 people, which did not vary by HL or DHL measures. The usage of online information sources varied by HL and DHL. The study findings can inform future student-focused interventions, including identifying the distinct roles of HL and DHL in pandemic information access, attitudes, and behaviors.

## 1. Introduction

The COVID-19 pandemic has been accompanied by a deluge of complex and changing information, resulting in an “infodemic”—an overabundance of information rife with misinformation and hoaxes [1,2,3]. Online communication channels are especially vulnerable to the spread of incorrect information, and many social media news feeds are refreshed automatically with similarly misleading content [1,2]. However, online communication channels have also been a central resource for reliable health information throughout the pandemic. The public is especially reliant on digital sources, given the restrictions on personal activities and face-to-face meetings [1].

Dealing with complex health information requires adequate health literacy [4,5,6]. As defined by Berkman and colleagues, health literacy (HL) is “the degree to which individuals can obtain, process, understand, and communicate about health-related information needed to make informed health decisions” [7] (p. 16). Digital health literacy (DHL) requires a unique skill set, including the ability to find, evaluate, appraise, integrate, and apply health information from online environments [8,9]. While the terms “eHealth literacy” (eHL) and “DHL” are often used interchangeably, eHL measurement is distinguished by its focus on online health information *gathering* [3,10]. DHL measures include a focus on interactivity on the Web, including adding self-generated content and protecting privacy [10].

Higher self-reported DHL has been associated with better health; more positive health behaviors, including prevention and management of chronic disease; and increased procedural health knowledge [10]. The need for skills in seeking, appraising, and applying digital health information is particularly critical for college students [10,11]. Young adults around the world often use the internet as their primary source of health information, spend much time online, utilize vast digital social media networks, and show trust in digital information [12,13,14]. American college students consider the internet to be their single most important source of health information [15]. Simultaneously, with multitudes of platforms, devices, and information streams, the digital landscape of health information has grown in complexity to the point where researchers struggle to assess young adults’ DHL [16].

In the United States (U.S.), roughly 17.5 million students attended college for the fall 2020 semester [17]. HL, DHL, and COVID-19-related attitudes and behaviors of college students have become a topic of increasing interest and relevance during the COVID-19 pandemic [18,19,20,21]. College students often live and interact in close quarters and travel long distances between home and school [14]. Students’ health-related decisions and behaviors impact COVID-19 infection rates, health outcomes, and the economic welfare of campuses and surrounding communities [22]. A better understanding of their health literacy skills and needs is essential to building useful programs, developing policies, and issuing health information across universities, health systems, and public health departments.

Although they are a relatively informed group on average, college populations still have limitations in HL and DHL [11,23]. These findings are troubling, as college students typically take increasing responsibility for their health and begin making more independent health- and safety-related decisions during this critical period [24]. A recent review found that many young adults suffer from low eHL, particularly in acquiring and vetting online information [11]. Other studies found that adolescents vary in their ability and confidence to appraise high-quality health information online [25,26]—a profound barrier in obtaining reliable health guidance. Recent research has also shown relationships between these skills and COVID-19 in college students outside of the U.S. A study of medical students at universities in Vietnam found that higher levels of HL were associated with less fear of COVID-19 compared to students with a lower level of HL [21]. A study on the DHL of university students in Germany and Portugal showed that although students generally achieve high DHL levels, they have difficulties making judgments about the reliability of online health information concerning COVID-19 [19,27].

It is essential to understand the HL and DHL of U.S. college students, along with their health profiles, patterns of health information-seeking behaviors and usage, and pandemic-related attitudes and behaviors. This inquiry can identify gaps in HL and inform future interventions. Moreover, HL and DHL skills are particularly important for college students in the U.S., given high infection rates, conflicting COVID-19 mitigation policies, a politicized information environment, and a lack of coordinated federal response and consistent public compliance [28]. Furthermore, best practices on interventions aiming to improve HL and DHL are limited, but existing research is promising [15].

Notably, while many of the definitions of HL and DHL focus on individual-level skills, there is a growing recognition of the social context in which health information is obtained and processed to build HL in general and specifically online [15,29,30]. Those with low HL and low DHL must find others to help them acquire and assess health information. Effective COVID-19 information dissemination strategies are needed for college students that leverage their social connectivity, especially in the digital environment. Evidence is necessary to guide intervention design for college-age focused interventions.

The study goal was to explore the associations of health information access and sources, as well as COVID-19-related attitudes and behaviors, with HL and DHL in U.S. college students. This study aims to contribute to the growing literature on DHL among college-age students. This study was conducted as part of the COVID-HL Consortium, an HL research network of 49 countries [20]. All country surveys contain common data elements, enabling the global monitoring of HL and allowing for interregional and international comparisons.

## 2. Materials and Methods

### 2.1. Sampling

Online survey responses (N = 256) were collected through a sequential sampling of a Qualtrics-managed research panel across the U.S. [31]. Prospective respondents were alerted of their eligibility for the questionnaire based on their system profiles. Individual invitations were sent electronically via Qualtrics.

The researchers used sampling quotas to mirror racial/ethnic and gender diversity in the national collegiate population as identified in the National Postsecondary Student Aid Study of 2016 [32]. Specific gender quotas ensured that at least 40% of the sample identified as female and at least 40% identified as male. Quotas also required at least 20% of respondents to be of Hispanic origin, 15% Black or African American, and 10% Asian. Responses were collected from July 7 to 23 July 2020.

Although the goal of this small study was not to represent the full U.S. collegiate population, we did want our distribution to be concordant with the national distribution on key elements. As we did not have strong a priori hypotheses about specific outcomes, nor were these COVID-19 outcomes well studied at the time of our sample, we chose a sample size that was adequate (β = 0.8) to identify differences of 20 percentage points in dichotomous outcomes for comparisons of low to adequate health literacy at α = 0.05 (N > 192). While the survey was active, the number of confirmed COVID-19 cases in the United States increased by 35%, from 2,984,541 to 4,022,985 [33].

Students received a benefit of 6 USD or less for participation, disbursed as digital gift cards or similar incentives by Qualtrics. The researchers performed quality checks using time metadata and free-response data to ensure participant consent, understanding, compliance, and good-faith reporting. The survey took about 10 min to complete. This project was confirmed as exempt from Human Subjects Review by the University of Hawai‘i Institutional Review Board.

### 2.2. Variables

The core survey battery followed instruments developed by the global COVID-HL Consortium [20], adapted slightly for American English. All responses were self-reported.

#### 2.2.1. Demographics

Respondents were asked about gender (female, gender variant or non-conforming, male, transgender female, transgender male); race (American Indian or Alaskan Native, Asian, Black or African American, Native Hawaiian or other Pacific Islander, two or more races, White); ethnicity (Hispanic, non-Hispanic); disability status (yes, no); first-generation college student status (yes, no); and political party affiliation (Democrat, Independent, Republican). This inquiry was included based on the politicization of the pandemic response in the U.S. [34]. Finally, race and ethnicity were combined into one variable (non-Hispanic Black, non-Hispanic Asian, non-Hispanic White, non-Hispanic other, Hispanic).

#### 2.2.2. Health Literacy

HL was measured with a modified version of the Single-Item Health Literacy Screener (SILS), designed to measure limited reading ability, a principal component of HL [35,36]. We chose this measure because the SILS has been validated as a reasonable measure to use in survey research [37,38]. This item asked, “How often do you need help to read instructions, pamphlets, or other written material from your doctor or pharmacy?” Responses included these choices: *Never*, *Rarely*, *Sometimes*, *Often*, and *Always*. Following the typical protocol for this commonly used instrument [35], the selection of *Sometimes*, *Often*, and *Always* indicated low HL, while responses of *Never* or *Rarely* indicated adequate HL.

#### 2.2.3. Digital Health Literacy

DHL was measured with an abbreviated version of the Digital Health Literacy Instrument (DHLI) used by the global COVID-HL Consortium [10]. The DHLI included 15 self-reported items with three questions in each of five modules designed to capture (a) information searching skills; (b) evaluating reliability; (c) evaluating relevance; (d) adding self-generated content; and (e) protecting and respecting privacy. We focused only on three subscales (searching, reliability, and relevance) as most relevant to our study. Subscale items were adapted to address the coronavirus topic specifically. For example, one question started, “When you searched the internet for information (for example, ‘Google’) about the coronavirus or related topics, how easy or difficult was it to…?” Each of three subscale items measured the skill on a four-point scale from *very difficult* to *very easy.* DHL was calculated for each subscale and the subscales’ average, which served as the primary DHL measure.

#### 2.2.4. Digital Health Information Sources

For planning future interventions, we also asked about the usage of 13 digital health information sources, including popular social media platforms and internet forums (e.g., TikTok, Facebook, YouTube, Instagram, Reddit).

#### 2.2.5. COVID-19 Attitudes and Behaviors

We assessed five sets of focal attitudes and behavioral responses to the pandemic: (a) response reaction; (b) vaccination; (c) public health guidance compliance; (d) likelihood to impact life; and (e) susceptibility. These were measured in a series of questions developed for this study by the COVID-HL Consortium [20].

First, respondents listed opinions on the public reaction: “All in all, do you think that the coronavirus outbreak has been made a bigger deal than it really is, made a smaller deal than it really is, or approached about right?” Next, students were polled on public health guidance compliance, including handwashing, social distancing, mask-wearing, and staying at home. These items were considered separately; then, responses were combined into a variable measuring full compliance (*yes* or *no* to all guidance). Respondents noted their likelihood to receive a coronavirus vaccination; the specific question asked, “How likely would you be to get a COVID-19 vaccine, if available?” Questions assessing susceptibility (“What are your chances of getting COVID-19”) and severity (“How would getting COVID-19 affect your life?”) were also included.

#### 2.2.6. Social Network

We assessed social network size with the following questions: “Over the past month, how many people did you discuss important matters of your health with?” and “Over the past month, how many people did you discuss important matters of their health with?” The topics of health discussions did not specifically need to be related to COVID-19. We also asked if respondents searched the internet for COVID-19 information for themselves and others.

### 2.3. Analysis

Descriptive data were considered as a whole and then by low and adequate HL and DHL levels. Descriptive comparisons of HL and DHL variables utilized Pearson’s chi-squared tests and analysis of variance (ANOVA) models as appropriate. We ran a multivariate logistic model predicting pandemic responses (attitudes and behaviors) with HL, DHL, and social network size, controlling for gender, race and ethnicity, disability, first-generation college student status, and political affiliation. Variables in the model were chosen to parsimoniously represent key predictive constructs to focal outcomes while minimizing the likelihood of multicollinearity. Finally, we considered descriptive information about digital health information sources by HL and DHL. A statistical significance level (alpha) of 0.05 and marginal significance level of 0.10 were set to identify, respectively, reportable and trend-level results worth further investigation. The analysis was completed with STATA 15, R 4.0, and Excel 2008 software packages.

## 3. Results

### 3.1. Demographics

Table 1 presents overall participant characteristics (N = 256). On average, respondents were 23.9 ± 4.3 years old; 108 (42%) identified as female, 140 (55%) identified as male, and 8 (3%) identified as one of the other gender options (e.g., transgender female, transgender male, gender variant or non-conforming). As designed, the sample ethnic and racial composition was generally representative of the U.S. college student population. Thirty-seven (14%) identified as Non-Hispanic Black, 24 (9%) as Non-Hispanic Asian, 91 (36%) as Non-Hispanic White, 98 (38%) as Hispanic, and 6 (2%) as a different racial/ethnic category. On average, students had matriculated 4.7 ± 3.7 semesters. One-hundred and forty-six (57%) respondents were first-generation college students (i.e., their parents and grandparents did not attend college). Respondents were split among political affiliations, with 132 (52%) students identifying as Democrats, 71 (28%) as Republicans, and 53 (21%) as Independents.

### 3.2. Health Literacy

More than half of this sample (51%, N = 130) reported low HL, as assessed by the modified version of the Single-Item Health Literacy Screener. Students with low HL were, on average, 2.3 years older than those with adequate HL (*p* < 0.001). Students who identified as female or gender variant were roughly twice as likely to have adequate HL than students identifying as male (*p* < 0.001). No significant differences between HL levels were found across ethnic or racial groups. Those with low HL were significantly more likely to be first-generation students (*p* = 0.047). There was no significant association between HL level and political affiliation.

### 3.3. Digital Health Literacy

The average score for DHL was M = 2.99 ± 0.51 on a scale of 1 (*very difficult*) to 4 (*very easy*). Roughly 57% of respondents indicated that it was *easy* to search, determine reliability, and establish relevance for information about the coronavirus or related topics, on average. DHL did not vary by HL level overall or for the three DHL subscales. Of the demographic variables, only disability status varied by DHL; those with a disability of any type reported lower DHL.

### 3.4. Digital Information Sources

**Figure 1:**
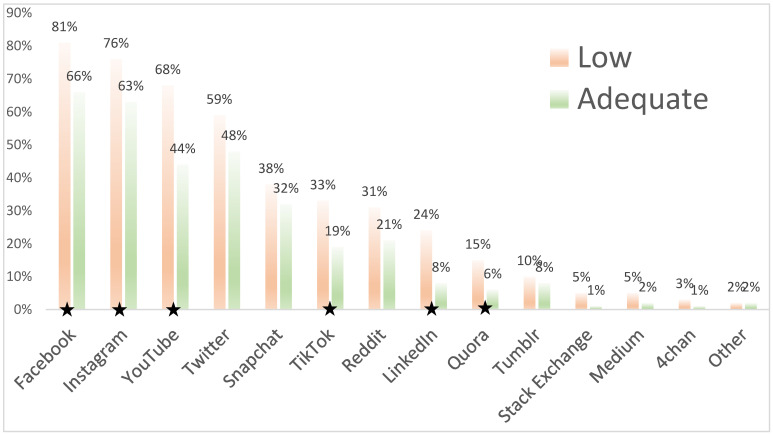
COVID-19 health information sources by health literacy. * Use of Facebook, Instagram, YouTube, TikTok, LinkedIn, and Quora all varied significantly at *p* < 0.05.

We asked college students about their social media use (Figure 1). Four platforms were utilized by more than half of the sample: Facebook (73%), Instagram (70%), YouTube (57%), and Twitter (54%). Students with low HL were significantly more likely to use all of these sources, except for Twitter. Those reporting low HL were more than twice as likely to use YouTube than those with adequate HL (*p <* 0.001). On smaller but increasingly relevant platforms, students with adequate HL were half as likely to seek pandemic health information on TikTok (*p =* 0.01) than their counterparts with low HL. Significant differences in DHL were found in two sources. Higher DHL was found in those who utilized LinkedIn (*p =* 0.008) and Tumblr (*p =* 0.02).

### 3.5. Attitudes and Behaviors

As shown in Table 2, many pandemic perceptions, attitudes, and behaviors differed by HL, DHL, or both. Seventy-three (29%) students felt that the public overreacted, 60 (23%) felt that the public underreacted, and 123 (48%) felt that there was a fair reaction. Students with low HL were twice as likely to perceive an overreaction than those with adequate HL (*p* = 0.008). Perception did not vary by DHL, however.

Approximately 43% of the sample believed that they had a low or no chance of getting COVID-19. This finding did not vary by HL level but did differ by DHL. Respondents who reported a lower chance of getting COVID-19 demonstrated higher DHL than those who acknowledged an elevated risk (M = 3.07 vs. 2.93; *p* = 0.04). If infected, 46% of the sample believed that COVID-19 would make them very sick. This finding did not vary by HL level but did differ by DHL. Those reporting that COVID-19 would make them very sick had higher DHL (M = 3.11) than those who responded that it would be not a big deal (M = 2.99) or would make them a little sick (M = 2.88) (*p* = 0.002).

Regarding prospective vaccination, 124 (48%) of respondents were somewhat or very likely to receive a COVID-19 vaccine if available. Planned vaccination behavior differed by the level of DHL but not HL. Those who were very likely to vaccinate reported higher DHL than those who were not as likely (3.07 vs. 2.92; *p* = 0.02). About 24% of the sample was compliant with all recommended public health behaviors. Compliance varied significantly by HL level. Thirty-one percent of those with adequate HL followed all compliance guidelines compared to 17% of those with low HL (*p* = 0.008). In addition, those who were always compliant had a higher average DHL (M = 3.22 vs. 2.92; *p <* 0.001).

### 3.6. Social Networks

On average, respondents spoke with 4.3 ± 5.0 people regarding any matter of their own health and 4.5 ± 5.8 others regarding their health issues. Social network size did not vary by HL or DHL. The majority of respondents reported using digital health information sources. Ninety-six percent of the sample had searched the internet for pandemic-related information within the past two weeks. Thirty-six percent sought information solely for themselves, 19% searched only for others, and 41% searched for both themselves and others.

### 3.7. Multivariable Models

Multivariable models predicting pandemic-related attitudes and behaviors by HL and DHL are summarized in Table 3. After controlling for gender, political affiliation, disability, first-generation college student status, and social network size, both HL level and DHL were independently associated with overall compliance with basic preventive practices (e.g., mask-wearing and social distancing). Higher DHL, but not HL, was significantly associated with greater willingness to get a COVID-19 vaccine, a belief that COVID-19 would likely be contracted, and a belief that infection would severely impact their life. Statistically significant (*p* < 0.05) variables included political affiliation with the Republican party in predicting perceived overreaction. Other variables showed marginally significant (*p* < 0.10) relationships in predicting COVID-19-related attitudes and behaviors. These included identifying as non-Hispanic Black in predicting the likelihood of vaccination and identifying as non-Hispanic Asian in predicting the perceived reaction level to the response. Social network size was not significant in predicting attitudes and behaviors.

## 4. Discussion

This study provides insights into HL and DHL levels, health-related attitudes and behaviors, and health-related social network use during the COVID-19 pandemic for a diverse sample of U.S. college students. Overwhelmingly, students utilize health information online and share it in a social context. In concordance with other literature, we find evidence that HL and DHL are related but unique constructs [10,39]. We provide new evidence of the relevance of this issue to pandemic-focused health information interventions and information sharing paradigms.

College students in this study had significant literacy challenges. Despite reasonably high educational attainment, over half of our sample self-reported low HL, supporting previous findings [23]. In addition, this study provides timely insights into the DHL of students amid university COVID-19 outbreaks nationwide. Adding to concern over low overall levels of HL, we find that the information environment is complicated. Many sources of digital health information are used, presenting opportunities for inconsistent or misleading health messages.

Despite the high prevalence of health misinformation on social media [40], many students are confident in their ability to navigate the digital health information landscape. Overconfidence about information may be a major issue; previous studies have shown a limited correlation between perceived skills and actual performance on Web-based health-related assignments [10,41]. Additionally, those with adequate HL showed more restraint in assessing their ability to verify source reliability, indicating that overconfidence may hinder adequate DHL.

Regarding pandemic responses, we found that HL and DHL were distinct and influential predictors. In multivariable models, both HL and DHL were independently associated with overall compliance with basic preventive practices (e.g., mask-wearing and social distancing). Higher DHL was associated with a willingness to be vaccinated against COVID-19. Moreover, those demonstrating lower HL levels were more likely to believe that the pandemic response was an overreaction. Perceived overreaction to public health crises combined with overconfidence in assessing health information may have compound negative effects and should be investigated further.

Social network size did not vary by HL or DHL, nor was social network size significant in the multivariable models predicting attitudes and behaviors. Nevertheless, the finding that college students had active health-related social network sizes of roughly five people is still important to note. The number of average members in these social networks was higher than in previous studies in more vulnerable groups [42,43,44]. On the one hand, a larger network size allows for a diversity of perspectives to inform decision making; however, five sources of health information may require significant individual skills and competency to unify varying—and sometimes contradictory—perspectives.

### 4.1. Implications

These findings support the growing call to make HL and DHL mandatory topics in the university curriculum, part of the university health communication strategy, or an extra-curricular course that students can participate in for free [45]. Promoting extant national health education standards in primary and secondary schools [46] and advocating for enhanced teacher training and education may support overall strategies to increase HL and DHL levels before students reach college. Students should also be taught critical thinking by educational institutions and how to discern accurate from misleading information. Empowerment is one of the main goals of health literacy.

At the tertiary level, administrators can also focus on approaches to strengthen individual HL and DHL, and information providers need to deal appropriately with false and misleading information, possibly through university-based monitoring and intervention systems for fake news (e.g., visual aids, seminars, social campaigns). Students with lower HL use social media more frequently and thus are at greater risk of exposure to false and misleading information, possibly becoming additional vectors in the propagation of poor information, encouragement of harmful health practices, or resistance to public health guidance. From an intervention standpoint, our findings suggest efforts not to increase the network size but engage the network in the distribution of accurate information.

As a complementary measure, universities can combat misinformation by starting social norms campaigns and using social marketing to disseminate “official” health information. While Facebook and Instagram were commonly utilized news portals, new platforms are being utilized and becoming increasingly important. TikTok was used by more than a quarter of our sample, which is notable given that it is arguably the youngest platform assessed in the study. Recent studies have identified TikTok as an innovative conduit to modernize the sharing of public health information while also acknowledging concerns about misinformation spread through this new platform [47,48].

### 4.2. Limitations

This study should be interpreted within the context of its limitations. Inherent limitations of an online survey include the potential exclusion of those with lower HL or DHL, those outside of the Qualtrics network, and those who lack access to the internet or requisite technology. Registration with a Qualtrics research panel requires intermediate computer skills, reliable internet access, and e-commerce ability. Furthermore, we focused on HL in a specific, relatively privileged population: U.S. college students. Educational level and income security are closely associated with compliance with COVID-19 public health mandates [49]. Another major limitation is the relatively small sample of U.S. college students. While the sample was representative of the racial/ethnic and gender diversity of the general student body, the small pool of respondents did not allow for other demographic characteristics, including socioeconomic status, to be taken into account. Moreover, the researchers did not analyze any potential moderating effect or association between the field of study and HL or DHL, including if the students were health sciences students.

The self-reporting nature of online surveys may have allowed respondents to respond inaccurately. The potential overestimation of health compliance should also be acknowledged. The modified SILS measures a singular, although significant, component of HL—self-reported reading ability. More comprehensive validated instruments, such as the European Health Literacy Survey Questionnaire and eHealth Literacy Scale, are used in corollary research studies, so results can be compared and contrasted [50,51,52]. Finally, as there is little information on college students’ social networks in the context of HL, we focused on the most basic metric: social network size. This selection limits the conclusions drawn about social networks’ role in health literacy and vice versa. Follow-up work should ask more detailed questions on information flow and relationships.

### 4.3. Future Research

U.S. college students are engaging with large social networks and sending and receiving health information from many sources. That said, our social network analysis did not examine reciprocity, closeness, frequency, or relationship (e.g., friend, family member, healthcare professional). These dimensions will be important areas for future research. Social context-focused interventions can provide opportunities for interventions that leverage positive influences and critical appraisal. Many students receive their health information through social media platforms: what they see on their feed is affected by others’ online behavior in their networks. This sequence may create opportunities for information, or misinformation, to spread quickly throughout a network.

This research aimed to inspire larger ventures in the spirit of the global COVID-HL Consortium, including studies of larger and more representative samples. More endeavors are needed that conduct comparative HL research, provide community-focused literacy programming, or highlight the value of HL in digital information navigation. DHL may play a different role across countries and settings, with differing patterns of social network influence and amounts of misinformation. Moreover, more consideration of social network types and composition will allow for a greater understanding of the interplay between digital health literacy and interpersonal sharing of health information.

## 5. Conclusions

An infodemic amid the COVID-19 pandemic places a tremendous burden on individuals, communities, and health systems. Given that students with adequate HL are more resilient to health misinformation and associated health conspiracy theories, increasing students’ HL and DHL will help combat negative health outcomes and the toxic effects of the current infodemic. The knowledge, attitudes, and behaviors of college students during the COVID-19 pandemic are of high interest because they seem to be factors that are intertwined with HL and DHL in the digital era. If universities commit to increasing health literacy in the student body, this could help innumerable people as students leverage their larger social networks to spread lifesaving information to those both within and outside the college setting.

## Figures and Tables

**Table 1:** General sample characteristics (N = 256).

Title	N	%
**Gender**		
Female	108	42%
Male	140	55%
Other Identity	8	3%
**Ethnicity and Race**		
Non-Hispanic Black	37	14%
Non-Hispanic Asian	24	9%
Non-Hispanic White	91	36%
Non-Hispanic Other	6	2%
Hispanic	98	38%
**Political Affiliation**		
Republican	71	28%
Democrat	132	52%
Independent	53	21%
**Disability**	49	19%
**First-Generation Student**	146	57%
**Searched Internet** **for COVID-19 Information**		
Yes, for me	92	36%
Yes, for others	48	19%
Yes, for me and others	106	41%
No	10	4%
**Health Literacy**		
Low	130	51%
Adequate	126	49%
	**M**	**SD**
**Age**	23.9	4.3
**Semesters Enrolled**	4.7	3.7
**Health Discussion Partners**		
Own Health	4.3	5.0
Their Health	4.5	5.8
**COVID-19 Digital Health Literacy (N = 246)**	2.99	0.51
Search Information Subscale	3.08	0.57
Determine Reliability Subscale	2.83	0.62
Establish Relevance Subscale	3.06	0.61

Note: Low health literacy was measured by reporting *Sometimes*, *Often*, and *Always* in the SILS, while responses of *Never* or *Rarely* indicated adequate health literacy. Rounding may result in percentage totals differing from 100%. M, mean. SD, standard deviation.

**Table 2:** Pandemic responses by health literacy and COVID-19 digital health literacy.

Title	Total		Health Literacy (N = 256)						COVID-19 Digital Health Literacy (N = 246)			
			Low		Adequate		Test				Test	
	N	%	N	%	N	%	χ^2^	P	$\bar{x}$	SD	F	P
**Total**	256		130		126			ns	2.99	0.51		
**Pandemic Perception**							9.55	0.008			0.08	0.92
Overreaction	73	29%	41	32%	32	25%			3.01	0.53		
Fair Reaction	123	48%	69	53%	54	43%			2.99	0.46		
Underreaction	60	23%	20	15%	40	32%			2.98	0.59		
**Vaccination Likeliness**							0.55	0.46			5.39	0.02
Very Likely	124	48%	60	46%	64	51%			3.07	0.54		
Somewhat/Not Likely	132	52%	70	54%	62	49%			2.92	0.47		
**Compliance**												
**Hand Washing**							9.76	0.002			17.08	<0.001
Yes	127	50%	52	40%	75	60%			3.12	0.50		
No	129	50%	78	60%	51	41%			2.86	0.49		
**Social Distancing**							2.60	0.11			8.71	0.003
Yes	119	46%	54	42%	65	52%			3.09	0.54		
No	137	54%	76	58%	61	48%			2.90	0.46		
**Mask-Wearing**							12.12	<0.001			9.53	0.002
Yes	166	65%	71	55%	95	75%			3.06	0.50		
No	90	35%	59	45%	31	25%			2.86	0.50		
**Staying at Home**							2.59	0.11			10.33	0.001
Yes	99	39%	44	34%	55	44%			3.12	0.50		
No	157	61%	86	66%	71	56%			2.91	0.50		
**Full Compliance**							6.94	0.008			17.50	<0.001
Yes	61	24%	22	17%	39	31%			3.22	0.48		
No	195	76%	108	83%	87	69%			2.92	0.50		
**Chance of Getting COVID-19**							1.51	0.22			4.31	0.04
No or Low Chance	110	43%	51	39%	59	47%			3.07	0.53		
Medium or High Chance	146	57%	79	61%	67	53%			2.93	0.48		
**COVID-19 Would Impact Life**							0.38	0.83			6.37	0.002
Not a big deal	17	7%	9	7%	8	6%			2.90	0.51		
Make me a little sick	121	47%	59	45%	62	49%			2.88	0.50		
Make me very sick	118	46%	62	48%	56	44%			3.11	0.49		

Note: Rounding may result in percentage totals differing from 100%; χ^2^, chi square. ns, not significant.

**Table 3:** Multivariable models predicting pandemic response.

Title	Pandemic Perception: Underreaction		Compliance with All Four Guidances		Very Likely to Vaccinate		Very Likely to Impact Life		High Chance of Getting COVID-19	
	OR	95% CI	OR	95% CI	OR	95% CI	OR	95% CI	OR	95% CI
**Low Health Literacy**	0.51 *	**0.24–1.04**	0.046 **	**0.23–0.92**	0.88	0.50–1.56	1.07	0.61–1.89	1.47	0.84–2.60
**COVID-19 Digital Health Literacy**	0.80	0.37–1.71	4.96 **	**2.39–10.26**	1.82 **	**1.04–3.19**	2.61 **	**1.48–4.60**	0.58 **	**0.33–0.99**
**Gender**										
Male (vs. Female)	0.73	0.35–1.54	0.74	0.35–1.53	1.21	0.66–2.22	1.45	0.79–2.66	1.08	0.59–1.98
Other Gender							5.09	0.73–35.38	6.11	0.67–55.45
**Race/Ethnicity**										
Hispanic	1.13	0.47–2.68	1.27	0.58–2.79	0.77	0.40–1.48	1.00	0.52–1.92	1.08	0.56–2.07
Non-Hispanic Asian	2.81 *	**0.94–8.36**	1.21	0.36–4.03	2.02	0.73–5.59	1.01	0.37–2.72	0.64	0.24–1.72
Non-Hispanic Black	0.40	0.11–1.49	0.91	0.30–2.82	0.41 *	**0.16–1.05**	1.31	0.54–3.20	1.21	0.49–2.96
Non-Hispanic Other	1.74	0.23–12.70	10.66	1.36–83.92	1.82	0.26–12.59	2.64	0.39–17.87	3.13	0.32–30.54
Non-Hispanic White										
**First-Generation Student (vs. not)**	0.74	0.37–1.51	0.69	0.36–1.33	0.77	0.44–1.34	1.26	0.73–2.19	0.74	0.43–1.29
**Disability (vs. no)**	0.80	0.29–2.23	1.21	0.49–2.98	0.88	0.43–1.79	1.12	0.55–2.27	1.13	0.59–2.31
**Political Affiliation**										
Republican	0.26 **	**0.09–0.69**	0.89	0.40–1.98	1.07	0.56–2.06	0.69	0.36–1.33	0.77	0.41–1.45
Independent	0.65	0.26–1.59	1.15	0.49–2.68	0.91	0.44–1.85	1.04	0.51–2.13	1.44	0.70–2.97
Democrat										
**Social Network Size**	1.04	0.37–1.50	1.02	0.96–1.09	0.99	0.92–1.06	0.97	0.92–1.03	1.01	0.95–1.08

Note: All “other gender” was one direction so was dropped from analyses (N = 8). CI, confidence interval. OR, odds ratio. ** *p* < 0.05 (statistical significance). * *p* < 0.10 (marginal significance/trend-level significance).

## Data Availability

Data will be made available upon request.
